# Clinical Significance of Achieving No Evidence of Disease in HER2-Positive Metastatic Breast Cancer: A Multicenter Study by Turkish Oncology Group (TOG)

**DOI:** 10.3390/cancers18060915

**Published:** 2026-03-12

**Authors:** Bengü Dursun, Şendağ Yaslıkaya, Serhat Sekmek, Tuğba Önder, Hasan Çağrı Yıldırım, Ömer Acar, Nadiye Sever, Savaş Gökçek, Sercan Ön, Ferit Aslan, Gül Sema Keskin, Zehra Sucuoğlu İşleyen, Gözde Savaş, Mehmet Emin Kalender, Semra Paydaş, Öznur Bal, Ülkü Yalçıntaş Arslan, Mustafa Şahbazlar, Burcu Çakar, Hacı Mehmet Türk, Hakan Akbulut

**Affiliations:** 1 Department of Medical Oncology, Faculty of Medicine, Ankara University, Ankara 06590, Turkey; hakan_akbulut30@yahoo.com; 2 Department of Medical Oncology, Faculty of Medicine, Çukurova University, Adana 01330, Turkey; drysendag@gmail.com (Ş.Y.); sepay@cu.edu.tr (S.P.); 3 Department of Medical Oncology, Ankara Bilkent City Hospital, Ankara 06800, Turkey; serhatsekmek@gmail.com (S.S.); oznurbal@sbu.edu.tr (Ö.B.); 4 Department of Medical Oncology, Dr. Abdurrahman Yurtaslan Oncology Training and Research Hospital, Ankara 06200, Turkey; ondertugba111@gmail.com (T.Ö.); ulkuarslan6@gmail.com (Ü.Y.A.); 5 Department of Medical Oncology, Faculty of Medicine, Hacettepe University, Ankara 06100, Turkey; hasancagri@windowslive.com; 6 Department of Medical Oncology, Faculty of Medicine, Manisa Celal Bayar University, Manisa 45030, Turkey; dr.acaromer@gmail.com (Ö.A.); m_sahbazlar@hotmail.com (M.Ş.); 7 Department of Medical Oncology, School of Medicine, Marmara University, Istanbul 34854, Turkey; dr.nadya@hotmail.com; 8 Department of Medical Oncology, Faculty of Medicine, Dokuz Eylül University, Izmir 35330, Turkey; gokceksavas35@gmail.com; 9 Department of Medical Oncology, Faculty of Medicine, Ege University, Izmir 35100, Turkeyburcucakar@gmail.com (B.Ç.); 10 Department of Medical Oncology, Medical Park Ankara Batıkent Hospital, Ankara 06370, Turkey; feritferhat2@gmail.com; 11 Department of Medical Oncology, Gülhane Training and Research Hospital, Ankara 06010, Turkey; gulsemayildiran@gmail.com; 12 Department of Medical Oncology, Faculty of Medicine, Bezmialem Vakıf University, Istanbul 34093, Turkey; zehrasucuoglu@gmail.com (Z.S.İ.); hmtuk@bezmialem.edu.tr (H.M.T.); 13 Department of Medical Oncology, Faculty of Medicine, Gazi University, Ankara 06560, Turkey; drgozdesavas@gmail.com; 14 Department of Medical Oncology, Meddem Hospital, Isparta 32040, Turkey; drkalender@hotmail.com

**Keywords:** no evidence of disease (NED), HER2-positive metastatic breast cancer, trastuzumab, pertuzumab, long-term responders, durable disease control

## Abstract

**Background**: Achieving no evidence of disease (NED) in HER2-positive metastatic breast cancer (MBC) has been proposed as a response-state marker for durable disease control. However, its clinical significance and predictive factors are not yet well established. **Methods**: In this multicenter retrospective study at 13 oncology centers in Turkey, we analyzed 118 patients with stage IV HER2-positive MBC who received trastuzumab-based first-line therapy and remained progression-free for at least 36 months. Patients were stratified by radiologically defined NED status according to RECIST v1.1. Clinicopathological features were compared between groups, and predictors of NED were identified using logistic regression. Progression-free survival (PFS) was estimated by Kaplan–Meier analysis and assessed with Cox regression. **Results**: Of the 118 patients, 55 (46.6%) achieved NED. Compared with patients who did not achieve NED, those attaining NED were younger (median 46 vs. 53 years, *p* = 0.013), had better ECOG performance status (*p* = 0.005), were more likely to have HER2 IHC 3+ status (*p* = 0.015), and higher histologic grade tumors (*p* = 0.013). In multivariable logistic regression, ECOG PS 0 (vs. 1 ; OR = 5.99, *p* = 0.005), HER2 IHC 3+ (vs. IHC 2+/ISH+; OR = 4.79, *p* = 0.035), and histologic grade ≤2 (vs. grade 3 ; OR = 0.25, *p* = 0.011) were independently associated with NED attainment. Patients who achieved NED had experienced significantly longer PFS than those who did not (median 131 months vs. 66 months; *p* < 0.005). In multivariate Cox regression, NED remained independently associated with prolonged PFS (HR = 0.24, *p* = 0.003). Metastasis-directed local therapy and treatment regimen (trastuzumab vs. trastuzumab + pertuzumab) were not independently associated with NED attainment or PFS. **Conclusions**: In this multicenter cohort of pre-selected long-term responders, nearly half of HER2-positive MBC patients achieved radiologic NED. Radiologic NED was independently associated with prolonged PFS within this enriched population. Factors associated with NED attainment included favorable ECOG performance status, HER2 IHC 3+ expression, and high tumor grade; given the wide confidence intervals, these estimates should be interpreted cautiously as exploratory and hypothesis-generating.

## Simple Summary

Although HER2-positive metastatic breast cancer is generally considered incurable, a subset of patients can experience durable disease control with anti-HER2 therapy. In this multicenter retrospective study, we examined the clinical significance of achieving radiologic no evidence of disease (NED) in patients who remained progression-free for at least 36 months after first-line trastuzumab-based treatment. Nearly half of these long-term responders achieved radiologic NED, and this status was associated with longer progression-free survival. Better performance status, HER2 IHC 3+ expression, and high-grade tumors were associated with a greater likelihood of achieving NED. These findings suggest that radiologic NED may be a clinically meaningful response state in selected patients with HER2-positive metastatic breast cancer. However, because this study was retrospective and restricted to long-term responders, the results should be interpreted cautiously and confirmed in prospective studies.

## 1. Introduction

Metastatic HER2-positive breast cancer (HER2+ MBC) has historically been associated with a poor prognosis and limited overall survival (OS). The advent of trastuzumab, however, marked a pivotal transformation, which has been further strengthened by the introduction of additional targeted therapies [1,2]. The CLEOPATRA trial established dual HER2 blockade with trastuzumab and pertuzumab plus docetaxel as the standard first-line treatment for HER2+ MBC, yielding substantial improvements in clinical outcomes [3]. Notably, with the pertuzumab-based regimen, 8-year follow-up data revealed that over a third (37%) of patients were alive and 16% remained disease progression-free [4]. These findings align with retrospective studies suggesting that durable, long-term responses are possible in select patients treated with anti-HER2 therapy—particularly those with hormone receptor-positive disease, oligometastatic presentation, surgical resection of metastases and/or the primary tumor, and no prior HER2-targeted therapy [5,6].

Attaining no evidence of disease (NED) has been linked to significantly prolonged progression-free survival (PFS) and OS [7,8]. Patients who achieve NED often present with solitary metastasis and are more likely to undergo surgical resection of the primary tumor or metastatic lesions [9,10]. Collectively, these observations suggest that radiologic NED may represent more than a transient therapeutic milestone, potentially serving as a robust response-state marker associated with durable disease control and prolonged PFS in patients with HER2-positive MBC.

The clinical significance of NED in patients with durable responses to anti-HER2 therapy remains unclear. Additionally, the clinical and pathological features associated with a higher likelihood of achieving NED are not fully defined. To address these uncertainties, this study compares the clinicopathological characteristics of patients with durable responses to anti-HER2 therapy by NED status, offering new insights into the role of NED in HER2-positive metastatic breast cancer.

## 2. Study Design and Patient Population

**Figure 1:**
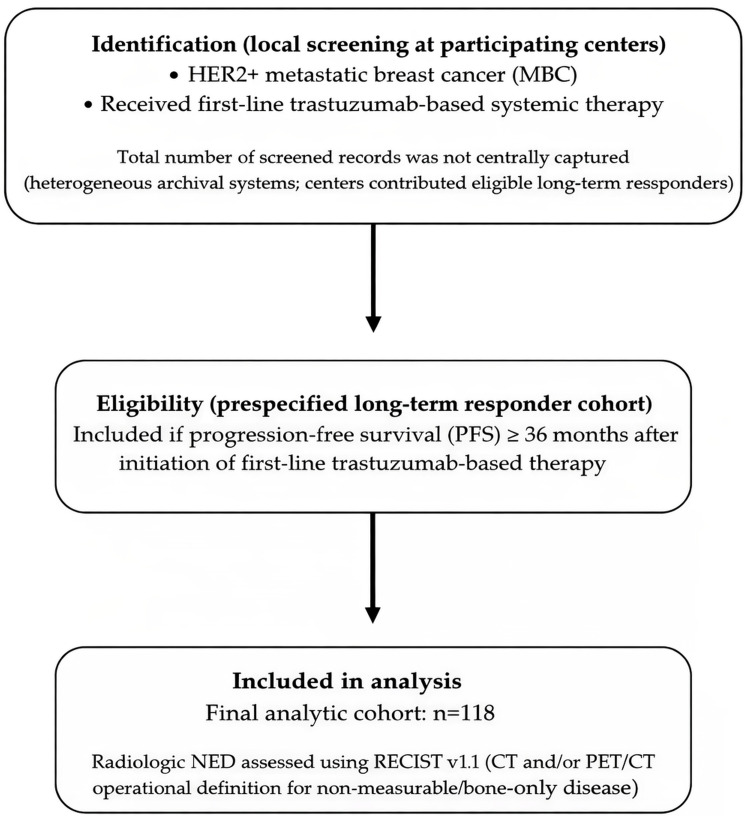
Cohort assembly flow diagram (CONSORT-like/STROBE-consistent).

This was a multicenter retrospective cohort study conducted in Turkey. Medical records of patients with stage IV HER2+ MBC diagnosed between 2012 and 2021 were reviewed. Eligible patients were required to have received first-line trastuzumab-based systemic therapy and to have remained progression-free for at least 36 months following initiation of treatment. Progression-free survival (PFS) was defined as the time from initiation of first-line anti-HER2 therapy to radiologically confirmed disease progression or death from any cause, whichever occurred first. Achievement of no evidence of disease (NED) was defined as the absence of detectable lesions on imaging (computed tomography and/or positron emission tomography–CT), using an operational definition of “radiologic NED” informed by RECIST v1.1 principles. Because “NED” is not a formal response category in RECIST v1.1, we used the term “radiologic NED” as an operational, clinically meaningful endpoint and applied RECIST v1.1 principles for its determination. We deliberately used the term “radiologic NED” rather than restricting the endpoint solely to RECIST CR because this real-world multicenter cohort included patients with bone-only and/or non-measurable disease, for whom a strict RECIST CR designation is not always directly applicable. For patients with measurable disease, NED corresponded to RECIST v1.1 complete response (CR), including resolution of target lesions and normalization of pathological lymph nodes (short axis < 10 mm). For patients with bone-only and/or non-measurable disease, NED required no radiologic evidence of active disease on CT and/or PET/CT and no new or progressive lesions; residual sclerotic bone changes without metabolic activity (when PET/CT was available) were not considered active disease. Bone scintigraphy was not used as a defining criterion for NED; when PET/CT was available, the absence of pathological FDG uptake was used to support inactive bone disease. All imaging assessments were performed as part of routine clinical care at participating centers and interpreted locally by experienced radiologists/nuclear medicine physicians; there was no central imaging review. Imaging modalities and assessment intervals were not standardized and reflected real-world practice at each center; therefore, potential inter-center variability and misclassification of radiologic NED status were acknowledged as limitations. A cohort assembly flow diagram is provided in Figure 1.

The following baseline characteristics were extracted: age at diagnosis, menopausal status, ECOG performance status (at initiation of first-line trastuzumab-based therapy), disease presentation (de novo vs. recurrent), metastatic sites (visceral vs. non-visceral, liver, lung, or central nervous system involvement), number of metastatic sites (1 vs. ≥2), HER2 status [IHC 2+/ISH+ vs. IHC 3+], hormone receptor (ER/PR) status, histologic grade, Ki-67, treatment regimen (trastuzumab alone vs. trastuzumab plus pertuzumab), concomitant endocrine therapy, and receipt of metastasis-directed local therapy (surgery, radiotherapy, or ablation). Patients were stratified into two groups according to disease status: those with NED and those without NED (non-NED). The primary aim of this study was to compare the clinicopathological characteristics of patients with durable responses according to NED status. The secondary objective was to evaluate PFS in relation to NED status.

This study was approved by the Institutional Review Board of the Clinical Research Ethics Committee of Ankara University School of Medicine [approval number: I04-262-23; Date of approval: 5 May 2023]. The requirement for informed consent was waived by the Ankara University School of Medicine Ethics Committee due to the retrospective design of the study. All methods were performed in accordance with the relevant guidelines and regulations, including the Declaration of Helsinki.

## 3. Statistical Analysis

Categorical variables were summarized as frequencies and percentages, and continuous variables as medians with ranges. Comparisons between patients with and without NED were performed using the chi-square or Fisher’s exact test for categorical variables and the Mann–Whitney U test for continuous variables. PFS was estimated by the Kaplan–Meier method and compared between groups using the log-rank test. Cox proportional hazards regression analyses were used to explore potential prognostic factors for PFS, while logistic regression analysis was applied to identify predictors of achieving NED status. Given the limited number of outcome events, covariates included in multivariable logistic and Cox models were selected a priori based on clinical relevance and the prior literature. Model estimates are reported as exploratory and hypothesis-generating. Radiologic NED was entered into the Cox model as a fixed covariate for pragmatic reasons; however, NED is a response state that can occur during follow-up and may be subject to guarantee-time (immortal time) bias when modeled as baseline. Therefore, Cox estimates for NED are reported as associative measures only. All statistical analyses were conducted using SPSS version 25.0 (IBM Inc., Armonk, NY, USA), and a two-sided *p*-value of <0.05 was considered statistically significant.

## 4. Results

In total, 118 patients with stage IV HER2+ MBC who had received trastuzumab-based first-line therapy and remained progression-free for at least 36 months were included in the analysis. All patients received trastuzumab as part of first-line treatment, and 60 (50.8%) also received pertuzumab, while none were treated with T-DM1. Within the study cohort, 55 patients (46.6%) achieved NED, whereas 63 (53.4%) did not. Compared with non-NED patients, those who achieved NED were significantly younger (median age, 46 years [IQR, 39–54] vs. 53 years [IQR, 43–61]; *p* = 0.013) and more likely to have a better performance status (81.8% vs. 55.6%; *p* = 0.005). HER2 IHC 3+ status was also more prevalent in the NED group (90.9% vs. 75.0%; *p* = 0.015). Notably, high-grade tumors (grade 3) were observed more frequently in patients who achieved NED compared with those who did not (66.7% vs. 38.9%; *p* = 0.013). No significant differences were found between the NED and non-NED groups regarding disease presentation (de novo vs. recurrent), menopausal status, site of metastases, presence of lung, liver or CNS metastases, use of metastasis-directed local therapy, treatment regimen (trastuzumab alone vs. trastuzumab plus pertuzumab), or Ki-67 expression (all *p* > 0.05) (Table 1). HER2 blockade was temporarily interrupted in 10 patients due to adverse events (median 4 months), with no difference between the groups (*p*: 0.24). 

In multivariable logistic regression, ECOG PS 0 (vs. 1 [reference]; OR = 5.99, 95% CI 1.72–20.79; *p* = 0.005) and HER2 IHC 3+ status (vs. IHC 2+/ISH+ [reference]; OR = 4.79, 95% CI 1.11–20.60; *p* = 0.035) were independently associated with higher odds of achieving NED, whereas histologic grade ≤2 (vs. grade 3 [reference]; OR = 0.25, 95% CI 0.08–0.72; *p* = 0.011) was associated with lower odds of NED attainment (Table 2). ER/PR status was also assessed in multivariable analyses and was not independently associated with radiologic NED attainment.

**Figure 2:**
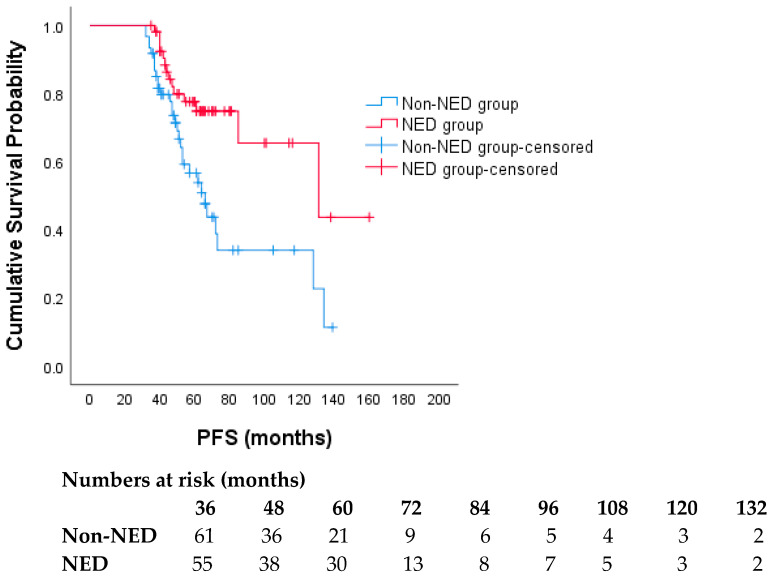
Progression-free survival according to NED status: Kaplan–Meier curves. Numbers at risk are shown below the plot. The cohort is restricted to patients with PFS ≥ 36 months at inclusion.

Disease progression was observed in 25.5% (14/55) of patients with NED, whereas 49.2% (30/61) of patients without NED experienced progression (*p* = 0.012). Patients who achieved NED had significantly longer PFS. Median PFS was 66 months (95% CI, 54.3–77.7) in the non-NED group, whereas it was 131 months in the NED group (95% CI, 52.0–209.9) (*p* < 0.005) (Figure 2). In the multivariable Cox regression, achievement of NED was the only independent predictor of prolonged PFS (HR 0.24, 95% CI 0.09–0.62; *p* = 0.003). After adjustment, ECOG PS, disease presentation, visceral involvement, number of metastatic sites, treatment regimen, HER2 status, tumor grade, hormone receptor status, age, and the use of metastasis-directed local therapy were not significantly associated with PFS (all *p* > 0.05) (Table 3).

## 5. Discussion

This multicenter study represents one of the largest real-world cohorts of patients with HER2+ MBC who achieved durable disease control following trastuzumab-based first-line therapy. Within this highly selected population, nearly half (46.6%) attained a state of NED, which remained independently associated with prolonged PFS. Notably, this rate is considerably higher than the ~16% reported in unselected MBC populations [11,12] but aligns with more recent analyses showing NED rates of 32–44% among long-term responders with HER2+ disease [13,14,15]. Taken together, these findings indicate that the probability of achieving NED has markedly increased in the era of trastuzumab-based anti-HER2 therapy. While NED-focused analyses have been reported previously, our study adds incremental value by leveraging a multicenter real-world cohort enriched for durable responders, thereby enabling estimation of the magnitude of the association between radiologic NED and long-term PFS in routine practice, and simultaneous assessment of clinicopathologic correlates of attaining radiologic NED within this trastuzumab-based treatment context.

Our study provides new insights by identifying NED as a clinically relevant milestone, associated with an estimated median PFS exceeding 10 years. Importantly, the median PFS estimates and the difference between NED and non-NED groups are valid only within our cohort, which was deliberately enriched by requiring PFS ≥36 months, and should not be extrapolated to unselected first-line HER2+ MBC populations. Considering the observation that 16% of patients maintained progression-free status at 8 years in the CLEOPATRA trial [4], this long-term benefit may largely reflect outcomes among those who attained NED [16,17]. However, because inclusion required patients to remain progression-free for ≥36 months, our cohort represents a highly selected group of long-term responders and is therefore subject to survivor (immortal time) bias, as patients who experience early progression or death are systematically excluded. Accordingly, the observed NED frequency and its association with prolonged PFS should be interpreted as correlates within this selected subgroup rather than as baseline predictive factors generalizable to the broader, unselected HER2+ MBC population at treatment initiation. Although long-term PFS has been associated with non-visceral disease, PR positivity, and HER2 IHC 3+ status, these associations were not evident in our cohort, likely reflecting restricted variability within this enriched cohort of patients with inherently durable responses. Consistent with prior reports, long-term PFS is most often observed in patients achieving complete response and receiving uninterrupted trastuzumab-based therapy [5,18]. In our study, treatment interruption occurred in only 10 patients, with a median duration of 4 months, whereas the majority continued therapy without interruption.

In the management of MBC, local therapy to the primary tumor and metastatic sites is being utilized with growing frequency, particularly among patients with oligometastatic or oligoprogressive disease [19,20]. Yet, there is no robust evidence supporting surgery or radiotherapy in metastatic breast cancer, as randomized trials—including those conducted across all subtypes rather than specifically in HER2+—have consistently failed to demonstrate a survival advantage [21,22,23]. Notably, metastasis-directed local therapy was not independently associated with NED status or PFS in our analysis. Importantly, by analyzing local therapy alongside systemic treatment variables in a multicenter real-world setting, we aimed to reduce confounding when interpreting radiologic NED; nevertheless, residual confounding cannot be excluded and reinforces the need to interpret NED primarily as a response-state marker rather than proof of eradication of systemic disease. Nevertheless, the prognostic interpretation of radiologic NED warrants caution and should not be equated with RECIST-defined CR or “cure”, particularly at early time points, because radiologic NED may be achieved after the addition of metastasis-directed local therapies that eliminate detectable lesions without necessarily reflecting durable systemic disease control. In clinical practice, early local therapy can result in radiologic NED yet still be followed by early progression due to occult micrometastatic disease; thus, the prognostic value of NED may be time-dependent. Given the retrospective nature and heterogeneity of our cohort, it is possible that selection bias and variability in practice patterns limited our ability to detect a survival effect of local therapy; moreover, information regarding whether local treatments were delivered with curative intent or solely for palliation was not available [24]. Accordingly, our negative finding regarding local therapy should be regarded as exploratory and non-confirmatory rather than definitive evidence of no benefit. Prospective studies are warranted to clarify the role of multimodal strategies in this setting. Future prospective studies incorporating standardized imaging schedules and time-dependent analytic approaches would also help define the dynamic relationship between local therapy, radiologic NED, and long-term outcomes.

Several baseline factors were associated with a higher likelihood of achieving NED, including younger age, favorable performance status, HER2 IHC 3+ expression, and high tumor grade. While HER2 overexpression has been consistently linked to greater sensitivity to trastuzumab-based therapy, the association of high histological grade with improved outcomes may appear paradoxical, as such aggressive features are generally correlated with poor prognosis [25,26,27]. We hypothesize that highly proliferative, HER2-driven tumors may be more susceptible to both cytotoxic chemotherapy and HER2-targeted therapies, resulting in deeper radiologic responses. Similar paradoxical associations between high-grade disease and treatment sensitivity have been reported in prior analyses [28,29,30]. Notably, the confidence intervals for some predictors were wide, reflecting statistical imprecision; therefore, these associations should be interpreted cautiously as exploratory rather than definitive.

### Methodological Considerations

The cohort was intentionally restricted to patients who remained progression-free for ≥36 months to characterize radiologic NED among durable responders in routine practice; however, this design uses a cohort enriched for favorable biology and introduces selection/survivor (immortal time) bias. In addition, radiologic NED is a time-dependent response state rather than a baseline characteristic; therefore, analyses treating NED as a fixed covariate should be interpreted as associative and hypothesis-generating, and future studies should apply time-dependent or landmark approaches in population-representative cohorts. Given the limited number of outcome events relative to candidate covariates, multivariable estimates may be imprecise and susceptible to overfitting, as reflected by wide confidence intervals. Descriptive data on the timing and duration of NED and relapse patterns were not uniformly available in this retrospective dataset; incorporating these measures in prospective studies would further clarify NED as a dynamic clinical state.

Despite the strength of a multicenter design and long follow-up, several limitations merit consideration. First, overall survival could not be assessed due to the low number of events, precluding evaluation of NED as a predictor of cure. Second, the retrospective design introduces inherent selection biases, particularly since all patients were required to remain progression-free for ≥36 months, thereby enriching the cohort for long-term responders. This design choice was deliberate to specifically characterize radiologic NED within durable responders; however, it limits inference regarding NED rates and predictors in unselected first-line HER2+ MBC populations and may partly explain differences from prior unselected cohorts. Third, radiologic NED is a time-dependent response state rather than a baseline characteristic; therefore, modeling NED as a fixed covariate may introduce guarantee-time (immortal time) bias and can inflate the apparent association with prolonged PFS. Accordingly, the reported effect estimates should be interpreted as associative and hypothesis-generating and validation using time-dependent or landmark approaches in population-representative cohorts is warranted. Because the number of outcome events was limited relative to the number of candidate covariates, our multivariable models may be subject to overfitting and unstable estimates; thus, findings should be interpreted as exploratory and require external validation. Fourth, treatment sequences did not include T-DM1 or newer HER2-directed agents (e.g., trastuzumab deruxtecan) and the 2012–2021 accrual period spans evolving standards of care, which may limit applicability to contemporary practice and influence deep response/NED rates. Additionally, patient-reported outcomes and the long-term burden/toxicity of chronic trastuzumab-based therapy (including cardiac monitoring) were not available in our retrospective dataset. Finally, imaging-based assessment of NED may not fully exclude minimal residual disease and heterogeneity in imaging modalities and assessment intervals across centers, and the absence of standardized criteria for therapy discontinuation in this setting limits the generalizability of findings.

## 6. Conclusions

In conclusion, our study shows that achieving radiologic NED under trastuzumab-based therapy is associated with durable disease control in HER2-positive MBC. In this selected cohort of long-term responders, radiologic NED appears to function as a clinically meaningful response-state marker; however, prospective validation in broader, population-representative cohorts and time-dependent/landmark analyses are required before considering NED a generalizable therapeutic endpoint. ECOG PS, HER2 IHC 3+ expression, and high tumor grade were independently associated with radiologic NED attainment in our multivariable models; nevertheless, the wide confidence intervals for some predictors indicate statistical imprecision, and these findings should be interpreted as exploratory and hypothesis-generating. Metastasis-directed local therapy was not independently associated with NED status or PFS in our analysis; however, this observation should be interpreted cautiously given the retrospective design, limited numbers, and likely selection biases. Future prospective studies integrating molecular profiling, contemporary HER2-targeted agents, standardized imaging schedules, and predefined local-therapy intent are needed to validate radiologic NED as a potential endpoint and to clarify its relationship with overall survival and long-term disease control in metastatic breast cancer.

## Figures and Tables

**Table 1:** Baseline clinicopathological characteristics of patients with and without no evidence of disease (NED).

Characteristics	NED (*n*: 55)	Non-NED (*n*: 63)	*p* Value
Age, years (median, IQR)	46 (39–54)	53 (43–61)	0.013
ECOG PS (0/1; unknown)	45/10; 0	35/26; 2	0.005
Disease presentation (de novo/recurrent; unknown)	36/19; 0	34/27; 2	0.34
Menopausal status (pre/post; unknown)	21/33; 1	33/27; 3	0.09
Site of metastases (non-visceral/visceral; unknown)	21/34; 0	19/42; 2	0.42
Lung metastasis (yes/no; unknown)	12/43; 0	17/42; 4	0.39
Liver metastasis (yes/no; unknown)	22/33; 0	21/40; 2	0.53
CNS metastasis (yes/no; unknown)	0/55; 0	2/58; 3	0.17
Number of metastatic sites (1/≥2; unknown)	14/41; 0	16/45; 2	0.92
Metastasis-directed local therapy (+/−; unknown)	14/41; 0	17/44; 2	0.76
Treatment regimen (T/TP)	23/32	35/28	0.13
Concomitant ET (yes/no; unknown)	34/21	45/15; 3	0.12
HER2 blockade interruption (yes/no; unknown)	3/51; 1	7/53; 3	0.24
HER2 status (IHC 2+/ISH+; IHC 3+; unknown)	4/50; 1	12/36; 15	0.015
ER status (+/−; unknown)	31/23; 1	34/14; 15	0.21
PR status (+/−; unknown)	19/34; 2	19/29; 15	0.83
Grade (≤2/3; unknown)	15/30; 10	22/14; 27	0.013
Ki-67 (≤20/>20; unknown)	12/35; 8	7/16; 40	0.66

ECOG PS: Eastern Cooperative Oncology Group performance status; ER: estrogen receptor; PR: progesterone receptor; HER2: human epidermal growth factor receptor; IHC: immunohistochemistry; T: trastuzumab only; TP: trastuzumab and pertuzumab; NED: no evidence of disease; OR: odds ratio. ORs compare the first category listed to the second (reference). The second category in parentheses is the reference group for all binary variables.

**Table 2:** Predictors of achieving NED status in logistic regression analysis.

Variable	Univariate OR (95% CI)	*p*-Value	Multivariate OR (95% CI)	*p*-Value
Age, years (≤40; >40)	0.59 (0.24–1.44)	0.25		
ECOG PS (0; 1)	3.34 (1.42–7.84)	0.006	5.99 (1.72–20.79)	0.005
Disease presentation (de novo; recurrent)	0.66 (0.31–1.40)	0.28		
Menopausal status (pre; post)	1.92 (0.91–4.05)	0.08	1.02 (0.35–2.97)	0.96
Site of metastases (non-visceral; visceral)	0.73 (0.34–1.57)	0.42		
Number of metastatic sites (1; ≥2)	0.66 (0.31–1.39)	0.28		
Metastasis-directed local therapy (yes; no)	0.88 (0.38–2.01)	0.76		
Treatment regimen (T; TP)	0.57 (0.27–1.19)	0.13		
HER2 blockade interruption (Yes vs. No)	2.24 (0.55–9.16)	0.26		
HER2 status (IHC +3/IHC 2+; ISH+)	4.16 (1.24–13.97)	0.021	4.79 (1.11–20.60)	0.035
ER status (positive; negative)	1.80 (0.79–4.10)	0.16		
PR status (positive; negative)	1.17 (0.52–2.62)	0.69		
Grade (≤2; 3)	0.31 (0.12–0.79)	0.014	0.25 (0.08–0.72)	0.011
Ki-67 (≤20; >20)	0.78 (0.26–2.36)	0.66		

ECOG PS: Eastern Cooperative Oncology Group performance status; ER: estrogen receptor; PR: progesterone receptor; HER2: human epidermal growth factor receptor; IHC: immunohistochemistry; T: trastuzumab only; TP: trastuzumab and pertuzumab; NED: no evidence of disease; OR: odds ratio. ORs compare the first category listed to the second category . The second category in parentheses is the reference group for all binary variables.

**Table 3:** Factors associated with PFS in multivariate Cox regression analysis.

Variable	Multivariate HR (95% CI)	*p*-Value
NED vs. non-NED status	0.24 (0.09–0.62)	0.003
Age, years (≤40; >40)	1.23 (0.44–3.42)	0.68
ECOG PS (1; 0)	1.31 (0.50–3.42)	0.58
Disease presentation (de novo; recurrent)	0.52 (0.19–1.38)	0.19
Site of metastases (visceral; non-visceral)	1.43 (0.52–3.92)	0.48
Number of metastatic sites (≥2; 1)	1.74 (0.65–4.65)	0.26
Metastasis-directed local therapy (yes; no)	0.74 (0.29–1.87)	0.52
Treatment regimen (TP; T)	0.62 (0.23–1.65)	0.34
HER2 status (IHC 3+ vs. IHC 2+; ISH+)	1.53 (0.48–4.85)	0.47
ER status (positive; negative)	1.07 (0.41–2.80)	0.88
Grade (3; ≤2)	1.95 (0.68–5.55)	0.21

ECOG PS: Eastern Cooperative Oncology Group performance status; ER: estrogen receptor; HER2: human epidermal growth factor receptor; IHC: immunohistochemistry; T: trastuzumab only; TP: trastuzumab and pertuzumab; NED: no evidence of disease.

## Data Availability

The datasets generated and/or analyzed during the current study are available from the corresponding author on reasonable request.
